# Book Review

**Published:** 1942

**Authors:** 


### Reviews of Books

**Medical Annual, 1942.** Edited by H. Letheby Tidy, D.M., F.R.C.P., and A. Rendle Short, M.D., F.R.C.S. Pp. lii., 396. Bristol: John Wright & Sons Ltd. Price 25s.—In spite of great difficulties the publishers have brought out their sixtieth volume in its usual form and with its usual content of up-to-date and stimulating information. In medicine one of the most interesting articles is that dealing with *Mass Radiology of the Chest.* The technique of the procedure is fully discussed and its value judiciously appraised. Modern views on *Blast injuries of the lung* are briefly but adequately summarized. On *Leishmaniasis* there is a useful reminder that the disease is more widely spread in Europe than is generally known, and is a common infection amongst dogs. The part played by the sandfly in transmission is emphasized. Treatment is well described. In the section on *War Psychiatry* the value of psychiatric examination of potential recruits and the assessment of their mental fitness are excellently dealt with. Much attention is given to *Scabies,* a disease which commonly increases in prevalence during wars. The article on *Burns of War* is particularly timely, though the description of the treatment of phosphorus burns is quite inadequate. *Diseases of the Cornea* contains some observations on the value of ascorbic acid in the treatment of corneal ulceration and superficial keratitis. The war-time increase in *Peptic Ulcers* renders the two articles on their medical and surgical interest exceptionally important. But there is something important and interesting in the *Medical Annual* for every variety of practitioner. Its quality and popularity continue undiminished.

**Materia Medica for Nurses.** By A. Muir Crawford, M.D., F.R.F.P.S.G. Fifth edition. Pp. viii., 138. London: H. K. Lewis & Co. Ltd. 1942.—That this book has reached a fifth edition in less than fifteen years since its first appearance shows that it is appreciated. This edition contains two new chapters, one on the Sulphonamide Group of Drugs and one on the D.D.A. and the Pharmacy and Poisons Act. The sections on General Anæsthetics, Hypnotics, Vitamins and Substances obtained from the Animal Kingdom have been enlarged. In the last-named chapter it is surprising to find no mention of protamine insulin without zinc, now usually called " Delay " insulin. In the vitamin section Riboflavin escapes mention, but these are unimportant omissions in a book for nurses, who will find it a readable and interesting source of instruction on drugs and their uses.

**Clinical Practice in Infectious Diseases.** By E. H. R. Harries, M.D., M.R.C.P., D.P.H., and M. Mitman, M.D., M.R.C.P., D.P.H., D.M.R.E. Pp. xii., 468. Illustrated. Edinburgh: E. & S. Livingstone. 1940. Price 17s. 6d.—Within its 456 pages, this book gives an excellent, up-to-date account of the infectious diseases of this country including prophylaxis. A novel but logical feature is the grouping of the hæmolytic streptococcal diseases, scarlet fever, erysipelas, etc., into one group. Another useful section is that dealing with the control of infectious diseases in hospital. The student and practitioner will find all he needs for modern methods of diagnosis and treatment in this volume. Owing to the large amount of facts compressed into a small compass, the style of the book is very impersonal and makes rather arid reading.

**Lectures on Conditioned Reflexes. Vol. 2.—Conditioned Reflexes and Psychiatry.** By I. P. PAVLOV. Translated and edited by W. H. GRANT, M.D., B.Sc. Pp. 200. Illustrated. London: Lawrence & Wishart Ltd. 1941. Price 8s. 6d.—Any paper from the Pavlov School of Physiology is worthy of attention and the present collection under review makes first-class reading. The range of subjects covered is very wide and includes many of contemporary psychiatric interest. Especially interesting is the chapter on the physiological explanation of hysteria, which is very relevant to war conditions. The translation is good and the book excellently printed. Altogether this book can be confidently recommended to anyone who appreciates the philosophic aspects of medicine, and who wishes to obtain an excellent grasp of contemporary neuro-physiology.

**Mule Spinners' Cancer.** First Edition. By E. M. BROCKBANK, M.B.E., M.D., F.R.C.P. Pp. 36. Illustrated. London: H. K. Lewis & Co. Ltd. 1942. Price 4s.—This little book, of thirty-six pages, gives a most interesting account of the disease, which has been recognized for about thirty-five years. Illustrations are given of the men at work, showing plainly why the left side of the scrotum is much more often affected than the right. The disease is not seen until the man has worked in the mills for at least ten years; there is usually some pre-cancerous condition of the skin such as papilloma or keratosis. It is due, of course, to the mineral lubricating oil used for the machine. Precautions recommended are a medical examination every six months, with excision of pre-cancerous patches; use of a less dangerous lubricating oil, and the application of a protective ointment.

**Surgery of Modern Warfare. Part I.** Edited by HAMILTON BAILEY, F.R.C.S. Pp. iv., 160. Illustrated. Edinburgh: E. & S. Livingstone. 1940. Price 12s. 6d.—This publication will be of great use to all who have to tackle the surgical problems of the present day. The many eminent contributors present the multiple phases of these problems with great clarity and maintain throughout an essentially practical approach. A particularly useful chapter is that on "Local Treatment of Infected War Wounds"; these are cases in which routine excision must not be carried out, and the amount of necessary interference is precisely outlined in tabular form. Another chapter deals clearly with all that is involved in excision of a fresh wound. The illustrations throughout are good, and the coloured plates are of particular excellence. As an up-to-date guide to treatment of air-raid casualties and of casualties in the forces this publication will be hard to excel, and we shall await the second part with more than ordinary interest; it is due to be published almost immediately.

